# Computational Requirements for Modeling Thermal Conduction in Polymeric Phase-Change Materials: Periodic Hard Spheres Case

**DOI:** 10.3390/polym16071015

**Published:** 2024-04-08

**Authors:** Kevin A. Redosado Leon, Alexey Lyulin, Bernard J. Geurts

**Affiliations:** 1 Mathematics of Multiscale Modeling and Simulation, Faculty EEMCS, University of Twente, P.O. Box 217, 7500 AE Enschede, The Netherlands; b.j.geurts@utwente.nl; 2 Group Soft Matter and Biological Physics, Department of Applied Physics and Science Education, Eindhoven University of Technology, P.O. Box 513, 5600 MB Eindhoven, The Netherlands; a.v.lyulin@tue.nl; 3 Multiscale Molecular Dynamics, Faculty EEMCS, University of Twente, P.O. Box 217, 7500 AE Enschede, The Netherlands

**Keywords:** conjugate heat transfer, high-fidelity simulation, effective thermal conductivity, OpenFOAM, resolution requirements, periodic systems

## Abstract

This research focuses on modeling heat transfer in heterogeneous media composed of stacked spheres of paraffin as a perspective polymeric phase-change material. The main goal is to study the requirements of the numerical scheme to correctly predict the thermal conductivity in a periodic system composed of an indefinitely repeated configuration of spherical particles subjected to a temperature gradient. Based on OpenFOAM, a simulation platform is created with which the resolution requirements for accurate heat transfer predictions were inferred systematically. The approach is illustrated for unit cells containing either a single sphere or a configuration of two spheres. Asymptotic convergence rates confirming the second-order accuracy of the method are established in case the grid is fine enough to have eight or more grid cells covering the distance of the diameter of a sphere. Configurations with two spheres can be created in which small gaps remain between these spheres. It was found that even the under-resolution of these small gaps does not yield inaccurate numerical solutions for the temperature field in the domain, as long as one adheres to using eight or more grid cells per sphere diameter. Overlapping and (barely) touching spheres in a configuration can be simulated with high fidelity and realistic computing costs. This study further extends to examine the effective thermal conductivity of the unit cell, particularly focusing on the volume fraction of paraffin in cases with unit cells containing a single sphere. Finally, we explore the dependence of the effective thermal conductivity for unit cells containing two spheres at different distances between them.

## 1. Introduction

The prediction of effective heat transfer is of great importance in a wide range of applications, particularly in the study of composite materials used for thermal energy storage (TES) [1]. TES refers to a system that stores heat energy for later usage and is based on the working principles of sensible and latent heat. A TES system is a sustainable energy solution that is commonly referred to as a ‘heat battery’. These systems play a critical role in efficiently storing and releasing thermal energy, thereby resolving the problem that energy is often generated and consumed at different moments in time [2].

The properties of composite materials can vary greatly due to their heterogeneous nature. In fact, the desired properties for TES materials are a combination of high thermal conductivity and a significant sensible and latent heat capacity. In this context, paraffin emerges as a particularly compelling candidate material because of its latent heat properties (150–250 kJ kg^−1^) [3,4] and widespread availability. Moreover, paraffin undergoes phase transitions within temperature ranges (0 to 90 °C) [3] that correspond closely to the requirements for domestic heating, thereby substantially enhancing its applicability [1]. However, the thermal conductivity of around 0.2 W m^−1^K^−1^ in the solid state, and even 0.08 W m^−1^K^−1^ [5] in the liquid state, would imply an impractically slow response to the loading and unloading of such a heat battery. Therefore, paraffin spheres encapsulated in polymer aerogels to prevent leakage [6], in combination with highly conductive nano-fillers, e.g., graphene [7,8], presents itself as a promising candidate composite material. With such materials, both a large storage heat capacity as well as high heat transfer rates may be achieved. These materials undergo a phase change as part of the heat storage process and will be referred to as phase-change materials (PCMs).

Accurately predicting the temperature distribution in structured heterogeneous materials is essential for understanding their functioning in heat batteries [9,10]. For system design, it becomes imperative to study the parameters governing heat transport on a small scale in a heat battery composed of paraffin for storage and nano-fillers for enhanced heat transport. We present the development of a fundamental simulation model and determine the spatial resolution requirements that should be met in order to achieve accurate predictions of the temperature distribution inside the material.

The analysis of the conjugate heat transfer (CHT) is challenging when aiming for an analytical temperature solution for complex heterogeneous media. In such cases, a numerical method is the only viable approach [11,12]. To understand the macroscopic heat transfer properties of a material, we will treat it as a continuum. Fourier first introduced the concept of thermal conductivity at a macroscopic scale [13], which led to the early development of Effective Medium Theory models by Maxwell-Garnett [14]. Numerous models to approximate the effective heat conductivity have been proposed since, which are either empirical, numerical or a combination of both [15]. However, these models often have unknown limitations. To overcome these uncertainties, our study uses a numerical approach to solve the complete underlying model formulated in terms of the governing partial differential equations.

Numerical discretization methods, including the finite volume method (FVM), finite element method (FEM) and finite difference method (FDM), are utilized to solve partial differential equations (PDEs). We adopt OpenFOAM [16,17], an open-source FVM computational fluid dynamics (CFDs) package. This simulation platform provides an option to add new solvers and post-processing to address specific problems specifically. This approach is suitable for the current heat battery study as it allows for proven numerical methods to be combined with tailored solutions to the problem of heat transfer in a configuration with multiple spheres in a temperature gradient.

This research focuses on numerically solving conduction-driven CHT in heterogeneous media composed of periodic configurations of stacked spheres of paraffin. The governing heat equations are discretized using OpenFOAM version 10 [16,17], where the use is made of the chtMultiRegionFoam solver, which provides the numerical solution to diffusive heat transport in domains containing various materials. The primary objective is to determine the accuracy with which the solution can be obtained and at what computational costs. In particular, the convergence of the solution upon refinement of the spatial grid is focused on. Ultimately, the rate of convergence obtained by OpenFOAM in multi-region simulations is determined and, correspondingly, the necessary spatial resolution needed to achieve a desired level of accuracy is quantified. By doing so, the reliability of the numerical approach for simulations of the heat transfer in stacked spherical particles is determined. This is crucial for the future investigation of heat conduction in genuinely complex systems, specifically focusing on the effective thermal conductivity (ETC) within spatially extended systems of randomly stacked spheres. The ETC of a one- and a two-sphere system per unit cell is determined to quantify the resolution requirements and specify the dependence of the effective conductivity on system parameters, such as the radius of the spheres and separation between spheres. The current investigations establish the feasibility and simulation conditions that should be adopted in simulations of more complex general configurations that are adopted to be determine the effective heat transfer in a so-called Representative Elementary Volume (REV), i.e., a unit cell for the periodic domain that contains a large number of spheres in an arbitrary configuration.

The numerical investigations have established that OpenFOAM can yield high-fidelity solutions, provided that a sufficient spatial resolution is employed. Achieving engineering accuracy, with errors in the temperature field within a few percent, necessitates approximately eight grid cells per diameter of the paraffin spheres. Nearly full convergence was attained with resolutions ranging from 32 grid cells per diameter and beyond. For these spatial resolutions, the approach displayed second-order convergence. A comparison between the ETC predicted by the Maxwell-Garnett model [14] and the numerical solution revealed close agreement up to a paraffin volume fraction of 30%. Beyond this volume fraction, gradual deviations were seen of 5–10%, e.g., at a 40–50% volume fraction. The successful modeling of the temperature field effects arising from the proximity of multiple spheres was achieved using OpenFOAM. The basic chtMultiRegionFoam solver was found to accurately predict the heat transfer and ETC in general unit cells with two paraffin spheres, including two overlapping as well as just touching spheres. This supports the potential extension of the simulation approach to configurations with multiple spheres per unit cell. The identified resolution requirement of eight grid cells or more per sphere diameter was seen to be sufficient even for configurations comprising multiple spheres.

The organization of this paper is as follows. In Section 2, the physical model and its mathematical formulation are introduced. The OpenFOAM implementation is described in Section 3. The simulation results specifying the temperature field and convergence upon grid refinement are discussed in Section 4. Finally, the concluding remarks are presented in Section 5.

## 2. Physical Model and Governing Equations

In this section, we first present the physical model of a heat battery in Section 2.1. The mathematical formulation of the governing equations is discussed subsequently in Section 2.2.

### 2.1. Physical Model of Heat Battery

**Figure 1:**
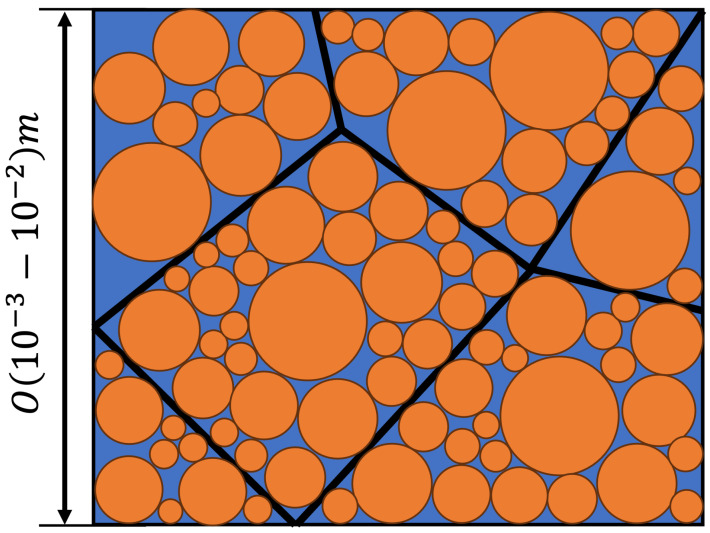
A two-dimensional representation of a TES microstructure composed of different size paraffin particles (orange) stacked in the pores of a foam (blue). The pores are bounded by a metal border, indicated symbolically by the thin black lines. In the actual porous metal foam, direct pathways connecting one pore with another are also contained—this is not included in the sketch.

The TES materials for phase-change materials (PCMs) exhibit diverse microstructures, designed to enhance heat transfer and facilitate rapid storage and release. The heat batteries that motivate the current study exploit a multiscale structure in which a large block of porous metal foam (*O*(10^−1^ m)) is used to transfer heat quickly and over comparably large distances. Within the pores (*O*((10^−3^–10^−2^) m) [10] of this foam, spheres of paraffin of a typical radius $r=O((10^{-5}–10^{-4}$) m) are stacked in a random configuration, available for sensible and latent heat storage. Figure 1 provides a 2D representation illustrating the stacked spherical particles of different sizes within the foam.

In Figure 1, it is apparent that a single pore comprises three materials with distinct properties. The paraffin spherical inclusions within the pore exhibit a low thermal conductivity of approximately 0.2 W m^−1^K^−1^ in the solid state and 0.08 W m^−1^K^−1^ in its liquid state [5]. This is tremendously small compared to, e.g., the copper from which the metal foam is composed, which has a thermal conductivity of around 400 W m^−1^K^−1^ [18]. Furthermore, the pore is saturated with still air, characterized by an even lower thermal conductivity of 0.0265 W m^−1^K^−1^ [19]. The thermal conductivity ratio of solid and fluid phases ($\kappa_{s}/\kappa_{l}$) for air-saturated metal foams is over 8000, indicating that the contribution of heat transfer by air can be largely neglected [19].

Motivated by Figure 1, we will consider approximate configurations to develop reliable computational methods for the simulation of the temperature field that develops when such a configuration is subjected to a steady temperature gradient (Dirichlet boundary) in the Z direction. To that end, we consider spatially periodic systems in the X-Y directions generated by repeating a suitable unit cell (periodic boundaries). In this paper, we focus on periodic unit cells with one or two paraffin spheres of the same diameter inside to study the numerical capturing of the effect of such inclusions. This generic problem corresponds to an approximate stacking of the spheres and enables precise numerical investigations.

After establishing the physical simulation domain, the PDE model for the heat transfer developing from a temperature gradient across the boundaries of the simulation box is specified next.

### 2.2. Mathematical Model

To accurately predict the thermal transport in composite domains, a CHT simulation provides a complete macroscopic model. This computational model enables the analysis of the contribution of conduction and convection mechanisms to the total heat transfer, consistently coupling all domains with appropriate interface conditions. We focus on process conditions that do not involve the melting of the paraffin—only heating and cooling are included at this stage. In this case, the heat battery problem considered needs to handle both the gaseous air (fluid domain) in the interstitial space left between the paraffin spheres, as well as the solid paraffin (solid domain). The system of partial differential equations (PDEs) governing the heat transfer in both the solid and fluid domains will be specified next. The numerical treatment of this model will be discussed in Section 3.1.

#### 2.2.1. Conservation of Energy

The basic principle of conservation of energy can be expressed concisely in terms of the evolution of the specific total energy (per unit mass) *e*. Closely following [20], we can include all the relevant mechanisms for our problem. Taking into account the pressure and shear forces, as well as the force of gravity as a body force, we may apply the Reynolds transport theorem to the fundamental first law of thermodynamics [20] and arrive after some simplification at:

(1) $$\frac{\partial (\rho e)}{\partial t}+\nabla \cdot \left(\rho ue\right)=-\nabla \cdot {\dot{q}}_{S}-\nabla \cdot \left(pu\right)+\nabla \cdot \left(\tau \cdot u\right)+\rho g\cdot u+{\dot{q}}_{V}$$

In this equation, u is the flow velocity, ρ denotes the material density, *e* stands for the specific total energy, *p* represents the pressure acting on the body, τ is the viscous tensor and g represents gravity. Additionally, ${\dot{q}}_{V}$ accounts for the heat generated or destroyed per unit volume, while ${\dot{q}}_{S}$ corresponds to heat transfer by diffusion, following Fourier’s law:

(2) $${\dot{q}}_{S}=-\kappa \cdot \nabla T$$

Here, κ denotes the thermal conductivity matrix, and ∇T represents the gradient of the temperature field *T*. The thermal conductivity matrix κ is a material property that for anisotropic media takes the form [21]:

(3) $$\kappa \left(T\right)=\left(\begin{array}{ccc} \kappa_{11} & \kappa_{12} & \kappa_{13}\\ \kappa_{21} & \kappa_{22} & \kappa_{23}\\ \kappa_{31} & \kappa_{32} & \kappa_{33} \end{array}\right)$$

To further understand the energy equation, we express the total energy in terms of the specific enthalpy, denoted as *H*, implying:

(4) $$e=H-\frac{p}{\rho }+\frac{1}{2}u\cdot u$$

where u denotes the velocity field describing the motion of the medium. Enthalpy for Newtonian fluids in thermodynamic equilibrium can be considered a function of the pressure and temperature, i.e., H=H(p,T), and can be evaluated using the standard equilibrium thermodynamic formula [22,23], which implies

(5) $$dH={\left(\frac{\partial H}{\partial T}\right)}_{p}dT+{\left(\frac{\partial H}{\partial p}\right)}_{T}dp=c_{p}dT+\left[v-T{\left(\frac{\partial v}{\partial T}\right)}_{p}\right]dp$$

Here, $c_{p}$ represents the specific heat capacity at constant pressure, and *v* denotes the specific volume. Equation (5) describes a chemically inert system of fixed mass [23]. Combining Equation (1) with Equations (4) and (5), we can formulate the energy equation for an incompressible fluid as:

(6) $$\frac{\partial }{\partial t}\left(\rho c_{p}T\right)+\nabla \cdot \left(\rho c_{p}uT\right)=\nabla \cdot \left(\kappa \cdot \nabla T\right)+{\dot{q}}_{w}$$

Equation (6) is a general equation where the source term ${\dot{q}}_{w}$ considers heating by shearing and pressure work. Equation (6) will be specified next for the solid and the fluid domain and solved later as a part of the total mathematical model.

#### 2.2.2. Heat Transfer in Solid Domain

When dealing with solid domains, Equation (6) can be significantly simplified as there is no material flow and the density remains relatively constant with respect to the temperature. We can simplify the equation as follows:

(7) $$\frac{\partial }{\partial t}\left(\rho c_{p}T\right)=\nabla \cdot \left(\kappa \cdot \nabla T\right)+{\dot{q}}_{V}$$

The thermal conductivity of paraffin was examined in [5,24] and observed to be nearly isotropic and homogeneous, with variations in the heat conductivity of up to approximately 20% over a very wide temperature range of 300 to 650 K. Therefore, we make the simplifying assumption that the storage material can be treated as isotropic, homogeneous and with material properties that are independent of the temperature. This assumption enables expressing thermal conductivity as κ=κI, where I is the identity matrix. The formulation in (7) can also be expressed in a non-dimensional form. In fact, upon introducing the reference time, length and temperature scales $\tau^{*}$, $L^{*}$ and $T^{*}$ and assuming that ρ, $c_{p}$ and κ are constant, we may write

(8) $$\frac{\partial T^{*}}{\partial \tau^{*}}=\nabla^{*2}\left(T^{*}\right)+{\dot{q}}_{V}^{*}$$

in case the time scale and the forcing scale are chosen as

(9) $$\tau^{*}=\frac{\kappa }{\rho c_{p}L^{2}}t\;\;\;;\;\;{\dot{q}}_{V}^{*}=\frac{\kappa (T_{hot}-T_{cold})}{L^{2}}{\dot{q}}_{V}$$

where *L* is the given characteristic length. It is convenient to impose standardized temperature boundary conditions if one defines the dimensionless temperature as

(10) $$T^{*}=\frac{T-T_{cold}}{T_{hot}-T_{cold}}$$

in terms of the temperatures $T_{cold}$ and $T_{hot}$ that define the temperature forcing of the system. Here, we use the same notation *T* for the dimensional and the non-dimensional formulation, as the difference is clarified by the context.

#### 2.2.3. Heat Transfer in the Fluid Domain

Spheres arranged in a stack within a pore in the metal foam are enclosed by air. This air also contributes to the overall heat transfer. We approximate the air in the interstitial volume as an incompressible fluid and consider convection and diffusion as driving mechanisms. Correspondingly, the dynamics are governed by the continuity equation, the conservation of linear momentum and the temperature equation as specified above. Because the flow of air between the randomly stacked paraffin spheres is on a very small scale and subject to a modest temperature difference on the scale of the diameter of an individual sphere, the heat transfer is dominated entirely by diffusion. We substantiate this simplification next.

The problem of heat transfer by the air between the paraffin spheres is governed by the Rayleigh number Ra [22], which characterizes the phenomena of heat transfer for natural convection. For values below a critical Ra number, heat is transferred primarily through thermal conduction and the effects of natural convection are considered negligible. Ra is defined as

(11) $$Ra=\frac{\beta g\Delta TL^{3}}{\nu \kappa }$$

Here, β, *g*, *L*, ν and κ represent the thermal expansion coefficient, gravitational acceleration, characteristic length, kinematic viscosity and thermal conductivity, respectively. Collecting typical values for these quantities [10], we observe the thermal expansion coefficient of air $\beta =3.5\times 10^{-3}$ K^−1^ [25]. Likewise, we recall that g≈10 m s^−2^ and take as the length scale for the interstitial air-filled domain the diameter of a paraffin sphere $L=5\times 10^{-6}$ m—this is likely to be an upper-bound for densely stacked spheres. The kinematic viscosity of air at room temperature is $\nu =1.5\times 10^{-5}$ m^2^ s^−1^ and the thermal conductivity can be estimated at $\kappa \approx 2.6\times 10^{-2}$ W m^−1^K^−1^. Adopting a very large temperature difference ΔT = 1 K over a distance of the radius of a sphere of approximately 5 μm, we may estimate $Ra=O(10^{-14})$, i.e., we infer that only dissipative heat transport is of relevance here. Even in the case where one would consider an empty pore in the metal foam with a much larger characteristic length of $L=5\times 10^{-3}$ m, the Rayleigh number is found to be $Ra\approx O(10^{-5})$, i.e., much lower than the critical $Ra\approx O(10^{2})$ for porous media [26]. Hence, the nonlinear convective transport is of little relevance here and diffusive transport dominates the heat transfer both in the solid and the fluid domain.

#### 2.2.4. Interface and Boundary Conditions

The heat transfer problem we consider here is characterized by interface conditions that ensure (i) the continuity of the temperature and (ii) the continuity of the heat flux across the interface. We discuss these conditions in more detail next:

(i) Continuity of temperature: There is no temperature jump at the interface, meaning that the temperature when approaching the interface from one side is equal to the temperature when approaching the interface from the other side, i.e.,
  (12) $${\left(T\right)}_{ij}\left(x^{*}\right)={\left(T\right)}_{ji}\left(x^{*}\right)$$
  where ${\left(T\right)}_{ij}$ is the temperature at any point $x^{*}$ on the interface between regions *i* and *j*, when approaching the interface from region *i*. Likewise, ${\left(T\right)}_{ji}$ is the temperature when approaching the same interface point $x^{*}$ from region *j*.
(ii) Continuity of temperature flux: This condition ensures that the total heat flux density is continuous when crossing the interface between regions *i* and *j*, at any location $x^{*}$. This condition takes into account the thermal conductivity of each region:
  (13) $${\left((\kappa \cdot \nabla T)\cdot n\right)}_{ij}\left(x^{*}\right)={\left((\kappa \cdot \nabla T)\cdot n\right)}_{ji}\left(x^{*}\right)$$
  expressing the continuity of the normal component of the heat flux density at any location on the interface between regions *i* and *j*, irrespective of whether the interface is approached from region *i* or region *j*. Here, n denotes the normal vector on the interface at $x^{*}$.

#### 2.2.5. Summary of Mathematical Model

The temperature distribution inside the domain consisting of air and paraffin is dominated by conduction in the parameter regime considered here. In the remainder of this paper, we will not consider explicit source terms. Hence, for region *i*, the problem is governed by:

(14) $$\frac{\partial }{\partial t}{\left(\rho c_{p}T\right)}_{i}\left(x\right)=\nabla \cdot {\left(\kappa \cdot \nabla T\right)}_{i}\left(x\right)$$

Periodic boundary conditions are used in the xy directions and the temperature is prescribed on the top and bottom of the unit cell in the *z* direction. Interface conditions with domain *j* are given by:

(15) $${\left(T\right)}_{ij}\left(x^{*}\right)={\left(T\right)}_{ji}\left(x^{*}\right)$$

for the continuity of the temperature and

(16) $${\left((\kappa \cdot \nabla T)\cdot n\right)}_{ij}\left(x^{*}\right)={\left((\kappa \cdot \nabla T)\cdot n\right)}_{ji}\left(x^{*}\right)$$

for the continuity of the heat flux across the interface.

## 3. Solver Description

In this section, we discuss the treatment of the governing heat equations in a finite volume framework as provided by OpenFOAM (Section 3.1). Moreover, the treatment of the adaptive meshing used for the accurate resolution of the finer details in the solution is presented (Section 3.2).

### 3.1. OpenFOAM Finite Volume Framework

OpenFOAM [16] is an open-source simulation platform for continuum mechanics. It utilizes the finite volume method (FVM) to discretize partial differential equations representing a wide range of physical phenomena [17]. We adopt OpenFOAM (version 10) in this study. OpenFOAM finds applications in diverse fields, including fluid dynamics, heat transfer and computational physics. By providing a comprehensive platform for numerical simulations, OpenFOAM enables the analysis of complex problems across various scientific and engineering disciplines.

**Figure 2:**
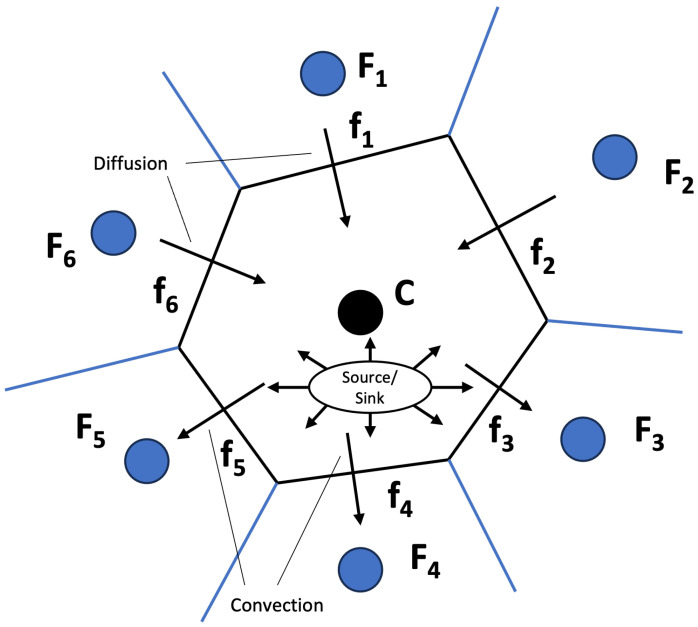
Conservation of a general scalar variable in a discrete element *C* of volume $V_{c}$.

The finite volume method (FVM) discretizes the computational domain into discrete control volumes, each representing a finite region within the domain. This numerical approach involves calculating fluxes across the faces of these control volumes and subsequently updating the values of the variables within each volume. In Figure 2, the conservation laws of a discrete volume $V_{c}$ and fluxes ($f_{i}$) crossing through the discrete element walls are illustrated. This process ensures adherence to conservation laws at the discrete level. Consideration of a conservation equation for a general scalar variable ϕ is expressed as:

(17) $$\underset{\mathrm{transient}\mathrm{term}}{\underset{︸}{\frac{\partial (\rho \phi )}{\partial t}}}\;+\underset{\mathrm{convective}\mathrm{term}}{\underset{︸}{\nabla \cdot (\rho U\phi )}}\;=\underset{\mathrm{diffusion}\mathrm{term}}{\underset{︸}{\nabla \cdot (\Gamma^{\phi }\nabla \phi )}}\;+\underset{\mathrm{source}/\mathrm{sink}\mathrm{term}}{\underset{︸}{Q^{\phi }}}\;$$

where $\Gamma^{\phi }$ represents the diffusion coefficient of the ϕ property. Dropping the transient term in Equation (17) to simplify our discussion on how the FVM discretizes, and integrating over the element the volume *V* of an element *C*, yields:

(18) $$\int_{V_{C}}\nabla \cdot \left(\rho U\phi \right)dV=\int_{V_{C}}\nabla \cdot \left(\Gamma^{\phi }\nabla \phi \right)dV+\int_{V_{C}}Q^{\phi }dV$$

Applying the divergence theorem to Equation (18) for both the convection and diffusive term and discretizing yields:

(19) $$\sum_{faces(V_{c})}\int_{f}\left(\rho U\phi \right)\cdot dS=\sum_{faces(V_{c})}\int_{f}\left(\Gamma^{\phi }\nabla \phi \right)\cdot dS+\int_{V_{C}}Q^{\phi }dV$$

where S is the surface vector. Equation (19) expresses the conservative nature of the method, emphasizing that a surface integral must be resolved along the faces that constitute the volume $V_{c}$ and a volume integral for the source $Q^{\phi }$. In the FVM, a Gaussian quadrature is employed to numerically evaluate the surface integral with the fluxes crossing and the volume integral.

**Figure 3:**
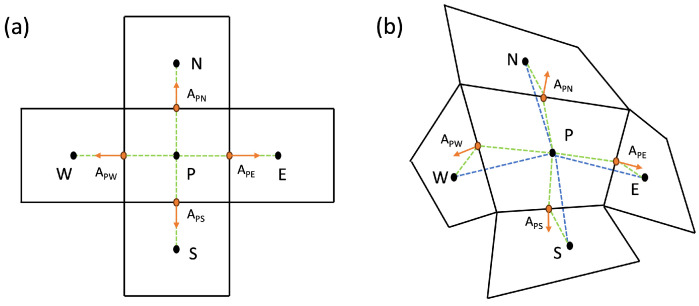
Cell in a structured uniform grid (**a**) and in a structured irregular grid (**b**).

Figure 3 presents a grid cell in a structure, distinguishing between a uniform and an irregular example. The temperature Equation (8) is discretized on this mesh arrangement with the key variables stored in cell centers and at cell interfaces. For simulations involving stacked spherical particles in a domain, an unstructured mesh is well suited for accurately representing the geometry and capturing the physics of the system.

The presence of multiple paraffin particles can be modeled in OpenFOAM by treating it as a multi-region case. OpenFOAM’s chtMultiRegionFoam solver is purpose-built for simulating CHT problems involving general configurations. This solver enables the accurate modeling of heat transfer phenomena across different materials and regions, irrespective of whether these contain a fluid or a solid.

In structured grids, cells are arranged regularly, often in a Cartesian fashion, simplifying the identification of neighboring cells and facilitating interpolation and flux calculations. This regularity is particularly advantageous for simulations involving simple geometries and where a regular mesh can be easily generated. In contrast, unstructured grid cells lack a regular arrangement, offering flexibility in mesh generation but requiring more sophisticated algorithms for interpolation and flux calculations due to irregular cell shapes. Unstructured grids are highly recommended for complex geometries and situations where mesh generation may be challenging with a structured approach, such as the case of paraffin inclusions. However, a noteworthy consideration in unstructured grids is errors in the calculations in cases of highly irregular cell shapes.

### 3.2. Meshing

This study focuses on the heat transfer simulation in a stack of spherical particles. The fidelity of the simulation results depends on the spatial resolution and the quality of the meshing of the domain. To achieve high-quality meshing, the snappyHexMesh mesh generation tool in OpenFOAM was adopted. This tool is designed specifically for generating hexahedral (hex) and prismatic (wedge) meshes, suitable for subsequent numerical treatment with any of the OpenFOAM solvers [16].

**Figure 4:**
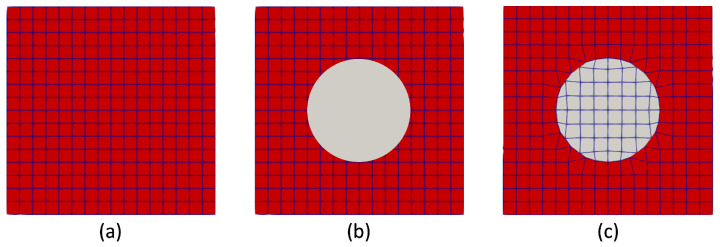
(**a**) Computation domain of structured cells constituting one region for the background mesh. (**b**) Structured background mesh with spherical inclusion surface. (**c**) New computational domain after snappyHexMesh consisting of two regions.

In the context of mesh generation using snappyHexMesh, an initial background mesh comprising hexahedral cells covering the entire computational domain is established. To maintain simplicity, a structured mesh serves as the starting background, offering a well-defined foundation. Following this, a complex geometry surface within the computational domain is added, along with its corresponding boundaries. The snappyHexMesh utility then identifies the features on the specified surface, initiating an iterative process where the cells surrounding the surface gradually conform to its shape. It refines the mesh around specified geometries and according to the size of gradients, as illustrated in Figure 4. The process starts with a base mesh, and refinement is applied around the edges of the given geometry.

During this iterative process, systematic mesh refinement is applied to the newly defined geometry region, guided by the number of cells in the background mesh. If necessary, additional refinement may be introduced by splitting cells in specified regions. Subsequently, the geometry region is removed from the background mesh and introduced as a new region with independent properties. This comprehensive process ensures the generation of a high-quality hexahedral mesh that accurately represents the complex geometry within the computational domain. Parameters governing the adaptation process include cell size, surface feature refinement level, and surface curvature. Properly setting these parameters is crucial for achieving an optimal balance between mesh resolution and computational efficiency.

## 4. Convergence of Temperature Predictions upon Grid Refinement

In this section, we explore the resolution requirements that need to be met for an accurate simulation of heat transfer in periodic domains. Such a system is generated by repeating a unit cell in all three directions. We consider two types of periodically extended systems: (a) containing a single sphere per unit cell and (b) containing two spheres per unit cell. Apart from this difference, we may also consider periodic systems with different volume fractions of spheres in the system. For a single sphere per unit cell, we assess the convergence of the temperature field in terms of temperature profiles across selected lines and the corresponding convergence of the L1-norm of the error. For systems with two spheres per unit cell, we investigate in addition the resolution needed to resolve the total temperature field even in the case of spheres separated by very small distances and even overlap.

### 4.1. Convergence Study Setup

For an accurate simulation of conjugate heat transfer in configurations of stacked particles, a spatial resolution analysis is essential to determine the number of grid cells per unit cell needed to achieve a certain accuracy level. The periodic setting of stacked spheres mimics more general configurations of such spheres within the pores of a metal foam, as described in the Introduction.

**Figure 5:**
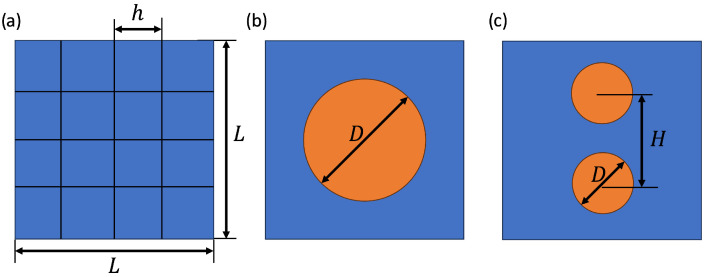
A two-dimensional representation of a periodic domain showing the following: (**a**) $n^{2}$ cells of *h* size composing a $L^{2}$ simulation box. (**b**) A single sphere with a diameter of *D* embedded within the simulation box. (**c**) Two spheres, each with a diameter *D*, whose centers are separated by a distance of *H*.

We detail the simulation setup in a 2D representation for clarity next. The actual simulations are all conducted in 3D. In Figure 5a, a 2D sketch of the simulation domain is shown, divided into a large number of grid cells. Each grid cell is of size $h^{3}$ where the grid spacing is taken uniformly as h=L/n, with *L* the size of the periodic unit cell and *n* the number of grid cells along each coordinate direction. Figure 5b presents a single paraffin sphere with a diameter *D* embedded in air, and Figure 5c depicts two paraffin spheres separated by a specified distance *H*. In the case of two spheres per unit cell, quite general configurations are possible. To limit the convergence study, we consider two spheres directly above each other, sensing maximal thermal gradients. Configurations with the spheres at general relative placement will have somewhat smaller thermal gradients, making these less demanding for our resolution study—these are therefore omitted here. Future work is planned on several spheres in general relative configurations—preliminary investigations show that the current OpenFOAM approach is capable of addressing these problems as well.

The vertical direction will be identified as the *z* axis. In the *z* direction, a temperature difference is imposed, characterized by a temperature difference ΔT over the distance *L*. The steady temperature field *T* in the entire domain, including the spheres, is simulated and the accuracy of the predictions is quantified. For this study, we refine the spatial resolution using $n=2^{k}$ grid cells per direction with k=1,2,…,7.

### 4.2. One Sphere per Unit Cell

The microstructure in the studied TES systems consists of multiple spherical particles of various sizes, densely packed together. To approximate the effective heat conductivity of such composite systems, we consider spatially extended periodic systems upon which a temperature gradient is imposed. In this subsection, we investigate periodic systems containing a single sphere per unit cell. This is a generic problem that is suitable to investigate numerical requirements for reaching a desired accuracy level.

**Figure 6:**
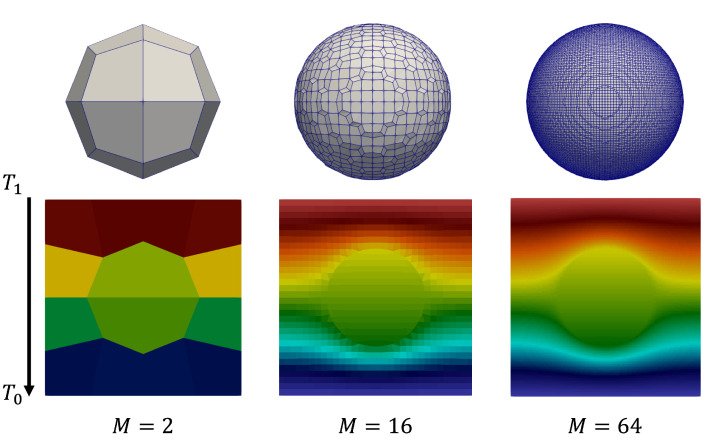
An embedded sphere in a periodic domain at different resolutions denoted by M=D/h. The predicted temperature fields *T* are shown below in terms of $T^{*}=\left(T-T_{0}\right)/\left(T_{1}-T_{0}\right)$ in which $T_{0}$ and $T_{1}$ are the imposed temperatures on the bottom and top, respectively.

**Figure 7:**
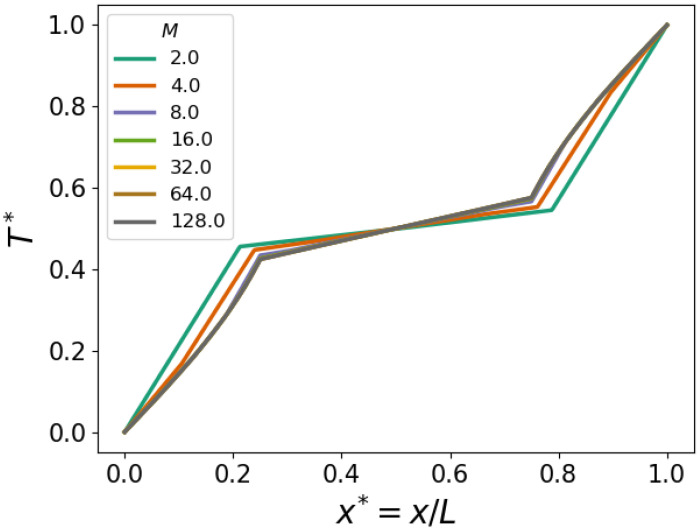
Temperature profile of sphere embedded in a cubic domain at different resolutions M=D/h.

The spatial resolutions that we consider are labeled in terms of the number of grid cells *M* that cover the diameter *D* of the sphere, i.e.,

(20) $$M=\frac{D}{L/n}=\frac{D}{h}$$

in terms of the domain size *L* and number of grid cells *n*. Figure 6 illustrates a cross-cut displaying various resolutions at different *M* values. A temperature gradient directed from top to bottom is imposed. The steady temperature fields show a clear qualitative convergence with increasing *M* with coarse structures recognizable even as M=2, as illustrated in Figure 7. This impression of convergence is quantified next.

**Figure 8:**
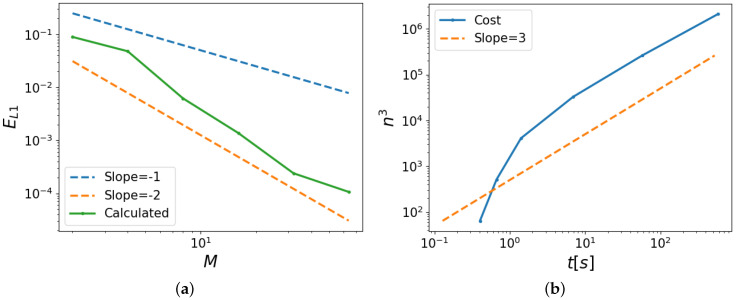
(**a**) The $L_{1}$—error for different spatial resolutions *M*. (**b**) The computational cost of the solver and SnappyHexMesh time against the total number of cells.

To assess the convergence of the temperature predictions, we compare as a function of *M* the dimensionless, scaled temperature

(21) $$T^{*}=\frac{T-T_{0}}{T_{1}-T_{0}}$$

where $T_{0}$ and $T_{1}$ are the imposed temperatures on the bottom and top of the periodic unit, respectively. We evaluate $T^{*}$ along a vertical line through the middle of the sphere. The corresponding temperature profiles are depicted in Figure 8 showing quantitative convergence with nearly grid independence in the case M≥8.

To facilitate a further quantitative analysis of the convergence, we assess the $L_{1}$—error in the predicted temperature profiles using the result with M=128 as the reference. We define the error as

(22) $$E_{L_{1}}\left(M\right)=\frac{1}{n(M)}\sum_{i=1}^{n(M)}\left|e_{i}\left(M\right)\right|$$

where n(M) denotes the total number of grid cells across the domain *L* at M=D/h. Moreover, the local error

(23) $$e_{i}\left(M\right)=T^{*}\left(z_{i}\left(M\right)\right)-T^{*}\left(z_{i^{*}}\left(M_{ref}\right)\right)$$

where $z_{i}\left(M\right)$ is the *i*-th grid point in the vertical grid corresponding to a selected value *M* and $z_{i^{*}}\left(M_{ref}\right)$ is the corresponding grid point at $i=i^{*}$ in the $M_{ref}=128$ grid in this study.

Figure 8a displays the convergence, indicating that indeed, for values of M≥8, convergence assumes asymptotic scaling equal to that of a second-order method. This was expected from the spatial discretization adopted in OpenFOAM employing Gaussian integration, which interpolates values from cell centers to face centers [16]. Finally, Figure 8b shows the increase in computational cost, which is defined as the time it takes for the complete mesh to be generated and the simulation to run, with increasing spatial resolution. We observe cubic scaling, verified by the power of three guiding lines.

In the next subsection, we consider the prediction of the temperature field in the case of two spheres per unit cell.

#### 4.2.1. Effective Thermal Conductivity

In the preceding subsection, we introduced a parameter for the spatial resolution, denoted as *M*, and observed consistent second-order convergence beyond a value of M=8.

The calculation of the effective thermal conductivity of the basic unit cell that contains the configuration of the paraffin spheres involves an examination of the overall thermal transport characteristics of the composite material, including its heterogeneity. In the numerical determination of ETC for a composite material, we assume steady conditions, which implies that the heat transfer across any plane at constant height *z* through the heterogeneous material remains constant. The total heat flow rate, denoted as $\dot{Q}$, flowing through a horizontal plane Γ at constant *z* is defined as follows:

(24) $$\dot{Q}=\int_{\Gamma }dxdy\kappa \left(x,y,z\right)\partial_{z}T\left(x,y,z\right)\equiv \kappa_{eff}\frac{\Delta T}{L}A$$

Here, κ represents the local thermal conductivity, and $\partial_{z}T$ is the temperature in the *z* direction evaluated plane. This expression also introduces effective thermal conductivity, $\kappa_{eff}$, including the temperature difference ΔT across the vertical length of the simulation box *L*, and the simulation box plane area *A*. By rearranging this expression, we obtain for the ETC:

(25) $$\kappa_{eff}=\frac{L}{A\Delta T}\int_{\Gamma }dxdy\kappa \left(x,y,z\right)\partial_{z}T\left(x,y,z\right)$$

In the steady state, the prediction of the ETC $\kappa_{eff}$ is independent of the particular plane considered. We may also use this property to verify the numerical evaluation of the ETC. For convenience and accuracy, we exploit this definition only at planes that traverse the domain through air. Independence was also established for planes that traverse both air and paraffin.

**Figure 9:**
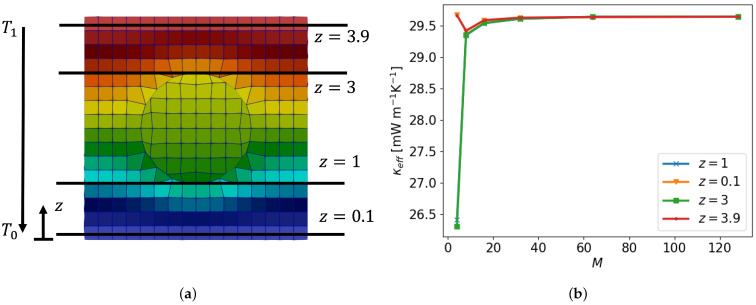
(**a**) Temperature field with an embedded sphere at M=16 with different sample planes (z=0.1, z=1, z=3 and z=3.9) to determine $\kappa_{eff}$ and (**b**) $\kappa_{eff}$ at different spatial resolutions *M* evaluated on different sample planes.

Examining the dependence of $\kappa_{eff}$ on the spatial resolution (*M*) is key for assessing what spatial resolutions are appropriate for reliable predictions. Figure 9 presents the temperature field (a) and the convergence of $\kappa_{eff}$ for a paraffin sphere with a thermal conductivity of $\kappa_{paraffin}$ = 0.2 W m^−1^K^−1^ in the solid state [5] and surrounding air with $\kappa_{air}$ = 0.026 W m^−1^K^−1^ [27]. The observed accuracy of predicting ETC aligns well with the convergence of the underlying temperature field established earlier. In particular, also for $\kappa_{eff}$, sensible predictions are found beyond M=8.

#### 4.2.2. Volume Fraction

Building upon the dependency of $\kappa_{eff}$ on the spatial resolution *M* outlined in the preceding subsection, we consider the dependence of the effective thermal conductivity on the volume fraction of paraffin. Various constitutive (micro-mechanical) models [28] have been devised to explore the effective thermal conductivity of composite materials, with the Maxwell-Garnett [14] and Bruggeman [29] models as prominent examples. For unit cells containing a single sphere, effective heat conductivity is given by:

(26) $$\kappa_{eff}=\kappa_{m}\left(1+\frac{3\phi (\delta -1)}{2+\delta -\phi (\delta -1)}\right)$$

The Maxwell-Garnett model, as depicted in Equation (26), offers a formulation for $\kappa_{eff}$ [14]. Here, $\kappa_{m}$ represents the thermal conductivity of the matrix material, while ϕ signifies the volume fraction of the filler material. The ratio between the thermal conductivity of the filler, denoted as $\kappa_{f}$, and that of the matrix is expressed as $\delta =\kappa_{f}/\kappa_{m}$. Notably, the Maxwell-Garnett model is tailored for spherical, non-overlapping particles, rendering it suitable for comparison with the simulation results.

**Figure 10:**
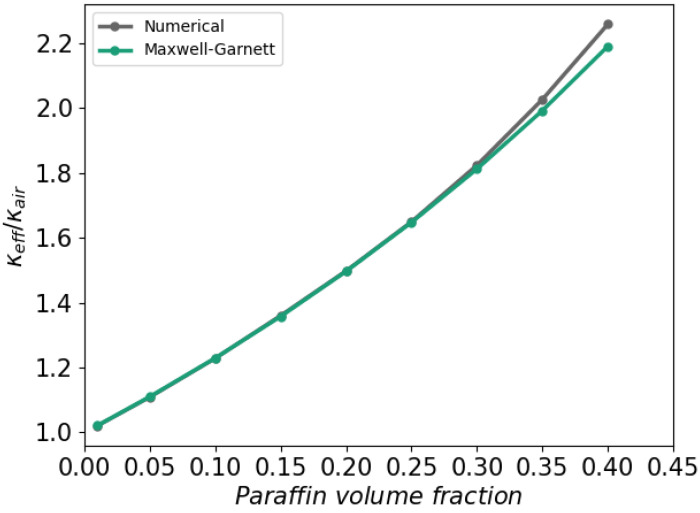
Effective thermal conductivity of binary mixture of still air and paraffin inclusions. The numerical approximation is compared with the Maxwell-Garnett model at a resolution of M=32 per sphere diameter.

Figure 10 presents a comparison between the numerical approximation and the Maxwell-Garnett model as a function of the volume fractions of the paraffin filler. Both approaches demonstrate a strong agreement, particularly up to a volume fraction of 30%. In these simulations, a constant spatial resolution of M=32 per sphere diameter is upheld; note that this implies a growing computational cost with a reduced volume fraction—this posed no feasibility problem using OpenFOAM.

### 4.3. Two Spheres per Unit Cell

To further investigate the predictions for the temperature field when using a periodic model for extended systems, we next investigate unit cells that contain two spheres. These spheres can be in any relative configuration inside the basic unit cell, which poses different challenges to the numerical method. We consider two extreme situations:

1. Horizontal. If the two spheres are aligned horizontally, i.e., the line through the centers of the spheres lies in a constant *z* plane, the temperature gradient experienced by the spheres would be quite similar to the temperature gradient experienced by a single sphere. This is particularly true if the two spheres are separated far enough, making their mutual interactions diminish. This situation was already studied in the previous subsection.
2. Vertical. If the two spheres are aligned vertically, i.e., the line through the centers of the spheres is in the *z* direction, the temperature gradient experienced by each of the two spheres differs most from the single-sphere case. Moreover, the gradients seen in this configuration are the largest among the different configurations. Therefore, this configuration will be studied in this subsection.

**Figure 11:**
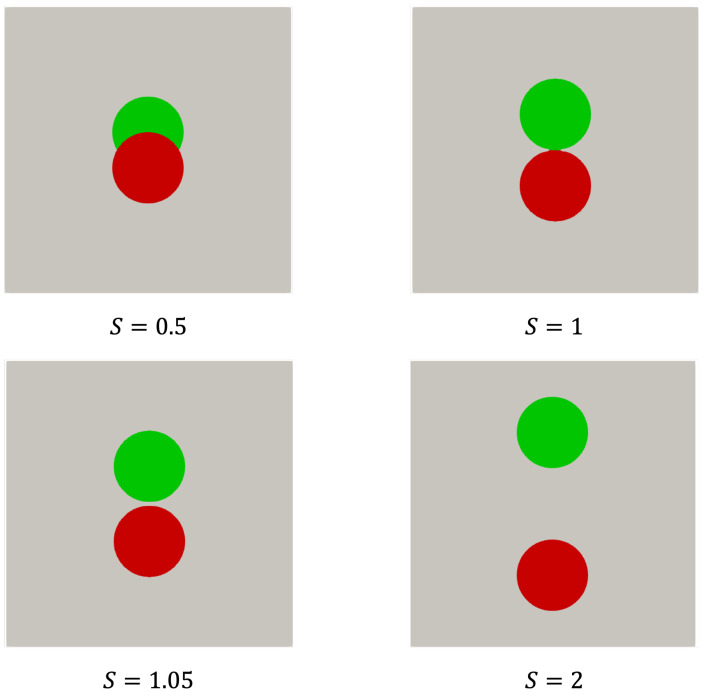
Two-sphere cases, illustrated as a red and green sphere of the same material, for different separations measured in terms of S=H/D, expressing the distance between the centers of the two spheres in units *D*.

Particles stacked within the unit cell may not always be well separated from each other. This mimics the situation when multiple spheres are stacked inside a pore of the metal foam in which a range of relative configurations may be expected. Therefore, we investigate the implications of different distances between the centers of the spheres on the predicted temperature field. We include overlapping, touching and separated configurations and consider the convergence of the corresponding solution upon grid refinement. Figure 11 illustrates four distinct cases, each characterized by different distance ratios S=H/D, measuring the distance between the centers of the spheres in units of the diameter of the spheres *D*, cf. Figure 5c. In terms of *S*, we observe that 0≤S≤1 corresponds to partially overlapping spheres and S>1 denotes separated spheres that, in principle, would allow for a grid fine enough to resolve the distance between the surfaces of the spheres with a number of grid cells.

**Figure 12:**
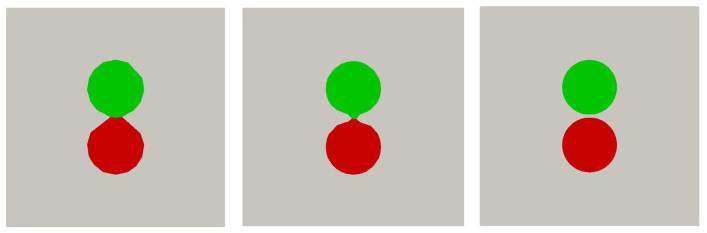
SnappyHexMesh refinement for spheres, illustrated as a red and green sphere of the same material, separated by a small gap (S=1.05) for different resolutions (M=4, M=8 and M=32).

Figure 12 illustrates two spheres separated by a small distance, corresponding to S=1.05. In case a coarse mesh (M=4) is used, SnappyHexMesh generates a computational grid that does not resolve the distance between the spheres but rather forms a dumbbell shape. As the spatial resolution increases, this artificial contact area diminishes until the gap is fully resolved at a sufficiently high resolution of M=32. Although there are clear differences in the way the geometry is resolved at different resolutions, the main question of course is how such differences affect the prediction of the temperature field. We turn to this next.

**Figure 13:**
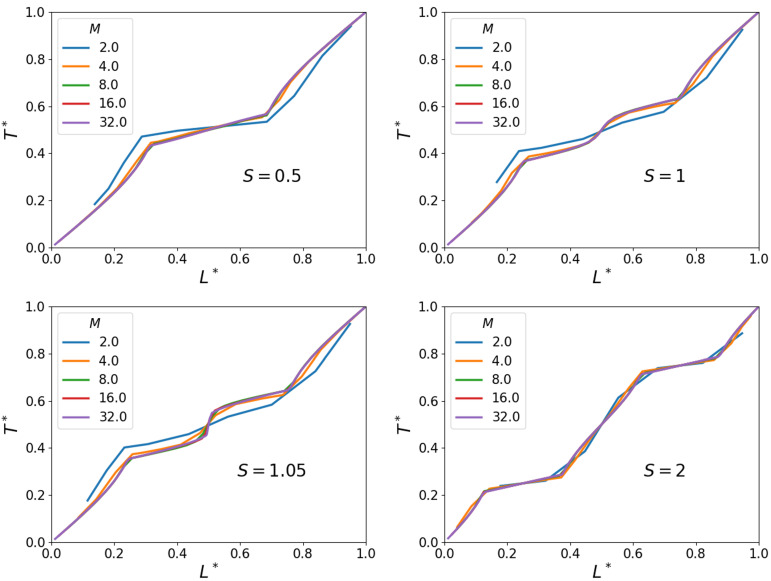
Vertical temperature profiles for various separations *S* and spatial resolutions *M*.

The cases depicted in Figure 11 (S=0.5,S=1,S=1.05andS=2) have been simulated at various resolutions *M*. The corresponding temperature profiles are presented in Figure 13. We observe a characteristic convergence of the temperature profiles with an increasing resolution as already presented for unit cells containing a single sphere only. Again, for M≥8, good general agreement with the grid-independent solution is observed, where it is understood that the value of *M* refers to the number of grid cells across the diameter of a sphere. This value of *M* also appeared for the single-sphere case, suggesting that the interaction between the spheres in terms of the spatial temperature distribution is rather modest and no particularly strong gradients emerge in the two-sphere configuration. Finally, for the case S=1.05 in which a small gap is present, even when not fully resolving the gap, the solutions are close to the fully resolved gap reference simulation.

#### Effective Thermal Conductivity

The study of the ETC of a two-sphere paraffin system embedded in air is considered next as a function of the distance (*S*) between the sphere centers.

**Figure 14:**
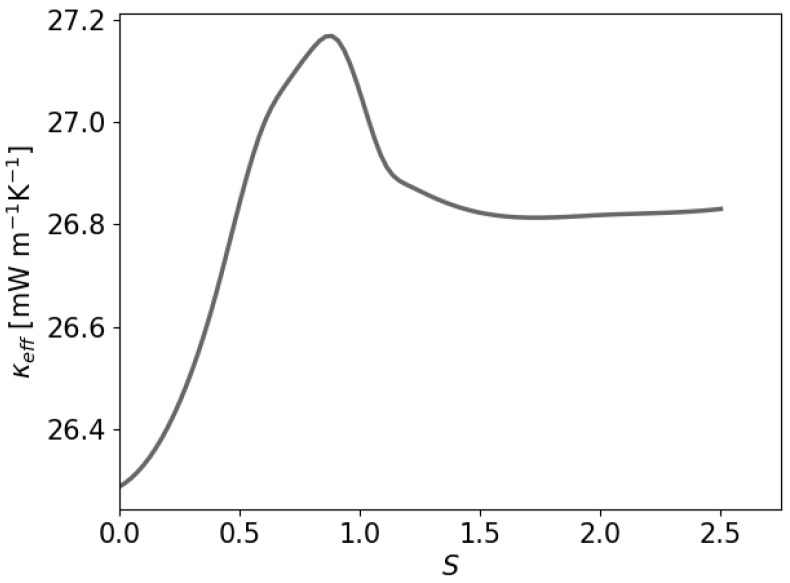
ETC of a vertically aligned two-sphere system at a spatial resolution of M=16 as a function of the distance *S* between the sphere centers.

Figure 14 displays the dependence of $\kappa_{eff}$ on the inter-sphere distance *S*. In this illustration, we used spheres of half the radius as used for the single-sphere case above. At S=0, the predicted value therefore does not agree with the single-sphere case considered earlier. The two-sphere systems show a value of $\kappa_{eff}$ = 0.0263 W m^−1^K^−1^ in the fully overlapping case at S=0. With increasing *S*, the effective heat conductivity increases and reaches a maximum near S=1. In this configuration, the path along which the heat is transported is for a large extent contained in paraffin for which the heat conductivity is larger than in the surrounding air. For larger separations, the value of $\kappa_{eff}$ reduces again to reach a plateau corresponding to two independent paraffin spheres. The simulation method appears to yield accurate predictions that can be used to define upscale theory as is considered in homogenization models.

## 5. Conclusions

In this paper, we developed a simulation method with which heat transfer in structured heterogeneous media can be simulated. The heterogeneous medium is meant to represent in detail the working material in a future thermal battery. Specifically, one may think of spheres composed of paraffin, although the method developed here is general. The working material contains a system of spheres placed in a certain configuration, which is repeated periodically. The corresponding unit cell can have several such spheres inside to represent actual stacked spheres in a realistic domain.

The approach is implemented in OpenFOAM, using second-order finite volume discretization. The full conjugate heat transfer problem of a periodic system was addressed, in which a unit cell is repeated indefinitely in all three directions, subject to a steady temperature gradient. The heat transfer in the case of a unit cell with only one sphere inside was considered in a grid refinement study. Visually, rapid convergence was appreciated upon increasing the spatial resolution, which could be recognized in detail to be of second-order accuracy. In fact, on grids with M≥8 grid cells per diameter *D* of the spheres, good engineering accuracy was observed, yielding high-fidelity results upon further refinement. The computational effort was seen to scale as $n^{3}$ where *n* is the number of grid cells in each coordinate direction. The computational effort is sufficiently low to enable the simulation of extensive periodic models of the composite material.

Further examples of this problem were investigated by considering unit cells with two spheres inside. Grid refinement showed second-order convergence also in this case. Moreover, in terms of the separation parameter S=H/D, we simulated two-sphere problems with overlap (0≤S≤1) as well as without overlap (S>1). Even in cases where possible small gaps between the two spheres would be smaller than the grid spacing *h*, the grid refinement showed continuous improvement upon increasing the resolution, with solutions that are very close to the eventual grid-independent solution. Hence, it appears that the under-resolution of tiny details in a complex stacking of spheres is not leading to large inaccuracies in the temperature field and the corresponding thermal transport.

The simulation method developed here was also illustrated in terms of the effective thermal conductivity $\kappa_{eff}$. We observed that at spatial resolutions M≥8 per sphere diameter, the effective conductivity can be computed reliably. This method can hence provide a basis for homogenization approaches to upscale the model to much larger systems. As an example, we calculated $\kappa_{eff}$ as a function of the volume fraction of paraffin filler and compared this with the Maxwell-Garnett constitutive model. The numerical approximation closely mirrors the Maxwell-Garnett model up to a 30% volume fraction of paraffin filler. This correspondence diminishes for yet higher volume fractions, as the periodic boundaries imply that the temperature distribution around the paraffin spheres can no longer be described as independent of that around nearby spheres. Our method was also adopted to compute the $\kappa_{eff}$ of a system of two spheres at different distances *S*. The two-sphere system revealed distinctive trends in the effective thermal conductivity. In fact, when going from overlapping to non-overlapping configurations, a peak ETC is observed slightly below S=1, attributed to the longer paraffin thermal pathway with a higher heat conductivity compared to the embedding air. These findings contribute to the data-driven upscaling of heat transfer models in truly complex systems of polymer composite materials.

The new model developed in OpenFOAM will be extended to systems with coated spheres, with which it will become possible to further improve the heterogeneous material by assigning the coating to increase the overall heat transfer rate and increase the loading and unloading of the core of the multiple spheres storing heat effectively in large quantities. Specifically, paraffin spheres coated with graphene form an important example of such composite materials. This type of extension is currently under investigation—the results will be published elsewhere. The application of the new approach to extended systems requires high-performance computations, which is well possible based on OpenFOAM. In fact, a possible grid of n=1024 and a resolution per diameter of M=8–16 would enable simulations of extended systems with $128^{3}–64^{3}$ spheres in a regular stack. This large-scale modeling forms the basis of future homogenization approaches that will enable the analysis of systems of realistic size and complexity.

Future research is devoted to effects due to variations in physical parameters, such as the volume fraction of the spherical inclusions. This aims to study the effect of changes in the physical system on the effective thermal conductivity. Additionally, the model will be extended by adding a thin coating composed of a material with very high thermal conductivity. A particular example would be the coating of paraffin spheres with graphene, thereby combining the fast and slow transport of heat in the system needed to realize particular designs for thermal batteries. Finally, after having established the resolution requirement for a single sphere and a pair of spheres, we will develop simulation methodologies that can handle large numbers of spheres (multi-spheres) touching each other. This would correspond closely to the situation motivated by Figure 1 and lead the way to realistic configurations. The detailed exploration and findings of these three studies will be presented elsewhere.

## Data Availability

The raw data from the simulations are available from K.A. Redosado Leon.

## Abbreviations

The following abbreviations are used in this manuscript:

CFD	Computational Fluid Dynamics
CHT	Conjugate Heat Transfer
ETC	Effective Thermal Conductivity
FEM	Finite Element Method
FVM	Finite Volume Method
PDE	Partial Differential Equation
PCM	Phase Change Material
TES	Thermal Energy Storage
REV	Representative Elementary Volume
